# Supplementary material for: Lipid biosynthesis enzyme Agpat5 in AgRP-neurons is required for insulin-induced hypoglycemia sensing and glucagon secretion
Source: Nat Commun. 2022 Sep 30;13:5761. doi: 10.1038/s41467-022-33484-6 (PMC9525695; doi:10.1038/s41467-022-33484-6)
Supplement: Supplementary file 1 — Supplementary Information [file 41467_2022_33484_MOESM1_ESM.pdf]

Supplementary Information

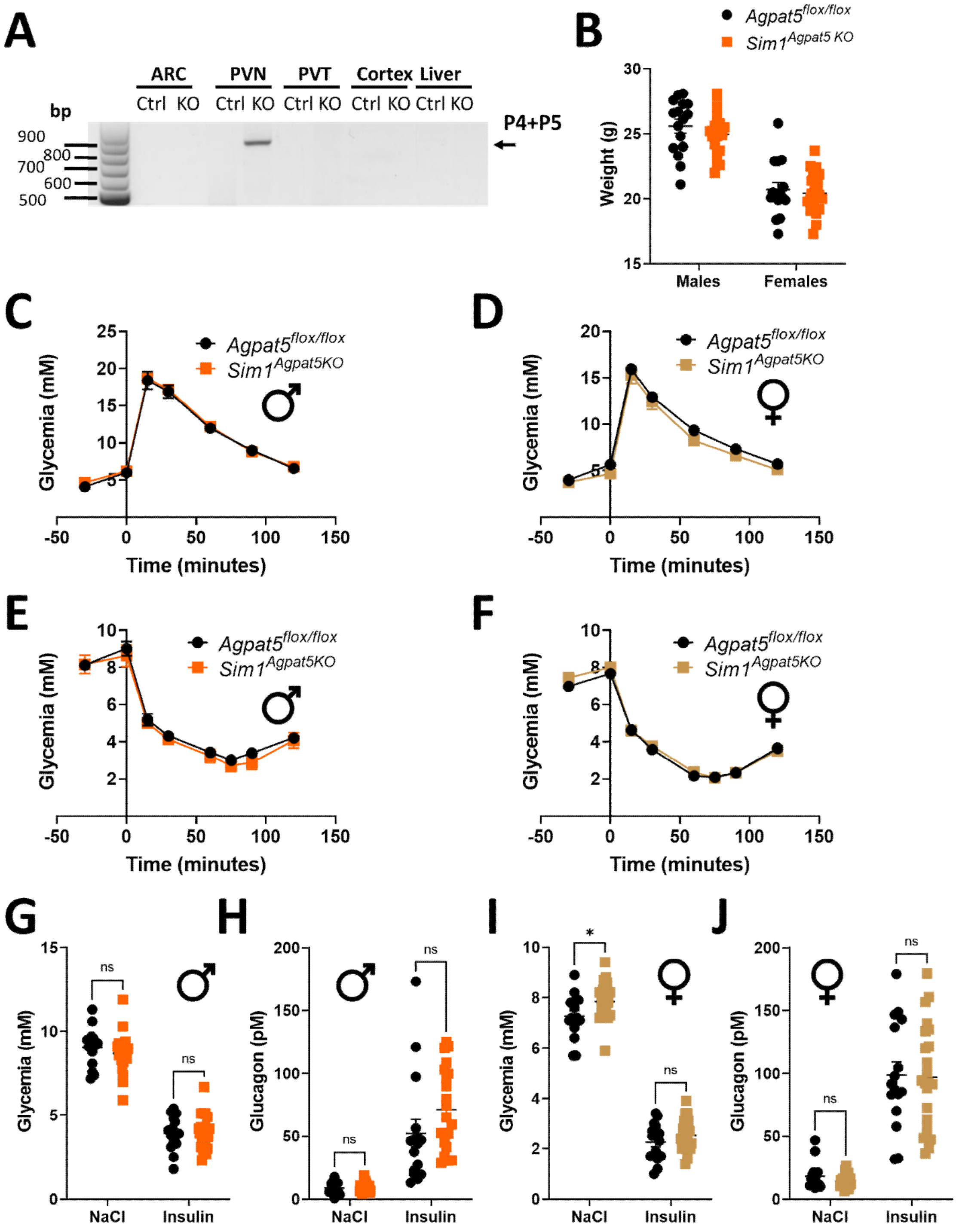

Supplementary Figure 1: *Agpat5* inactivation in Sim1 neurons has no effect on glucagon secretion in male and female mice

(A) PCR analysis of *Agpat5* recombination in micro-dissected PVN from *Agpat5$^{flox/flox}$* (Ctrl) and *Sim1^{Agpat5KO}* (KO) mice. Representative image is presented.

(B) Body weight of male and female *Agpat5*$^{flox/flox}$ and *Sim1*^{Agpat5KO} mice,

(C) Glucose tolerance tests in male mice.

(D) Glucose tolerance tests in female mice

(E) Insulin tolerance tests in male mice.

(F) Insulin tolerance tests in female mice.

(B-F) n=8-10 mice. Data are mean±SEM. Two-way ANOVA (repeated measurements) with Sidak's multiple comparisons correction.

Glucagon plasma levels one hour following saline or insulin injections in male and female *Agpat5*$^{flox/flox}$ and *Sim1*^{Agpat5KO} mice

(G) Glycemia of male mice.

(H) Plasma glucagon in male mice.

(I) Glycemia of female mice.

(J) Plasma glucagon in female mice.

(G-J) Two independent cohorts, n=10-14 mice per genotype per cohort. Data are mean±SEM. *$p<0.05$, two-way ANOVA with Tukey's post hoc test.

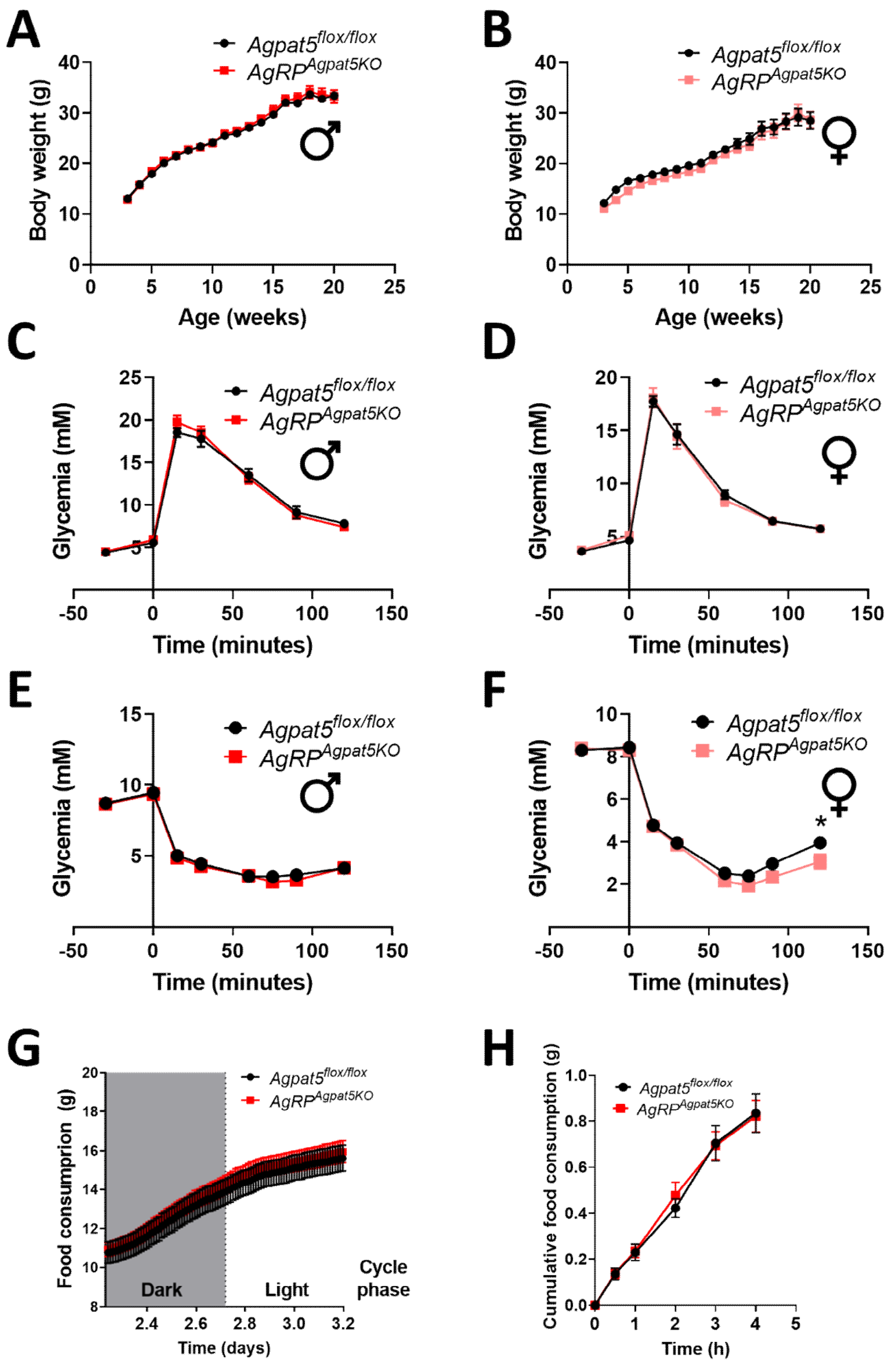

Supplementary Figure 2: Inactivation of *Agpat5* in AgRP neurons does not affect body weight, glucose tolerance, and insulin tolerance in male and female mice

(A, B) Body weight progression between 3 and 20 weeks of age in male and female *Agpat5*$^{flox/flox}$ and *AgRP*^{Agpat5KO} mice, respectively. Data are mean±SEM, n=8-13 mice per genotype.

(C-D) Glucose tolerance test in males and females.

(E-F) Insulin tolerance tests.

(C-F) Two independent cohorts, n=7-14 mice per genotype. Data are mean±SEM. *p<0.05, two-way ANOVA (repeated measurements) with Sidak's multiple comparisons correction.

(G) Food intake in male *Agpat5*$^{flox/flox}$ mice and *AgRP*^{Agpat5KO} mice over 24 h period with ad libitum food.

(H) Cumulative food consumption in 4h of refeeding after 16 h fasting period in male mice.

n=10-12 mice per genotype. Data are mean±SEM. *p<0.05, two-way ANOVA (repeated measurements) with Sidak's multiple comparisons correction.

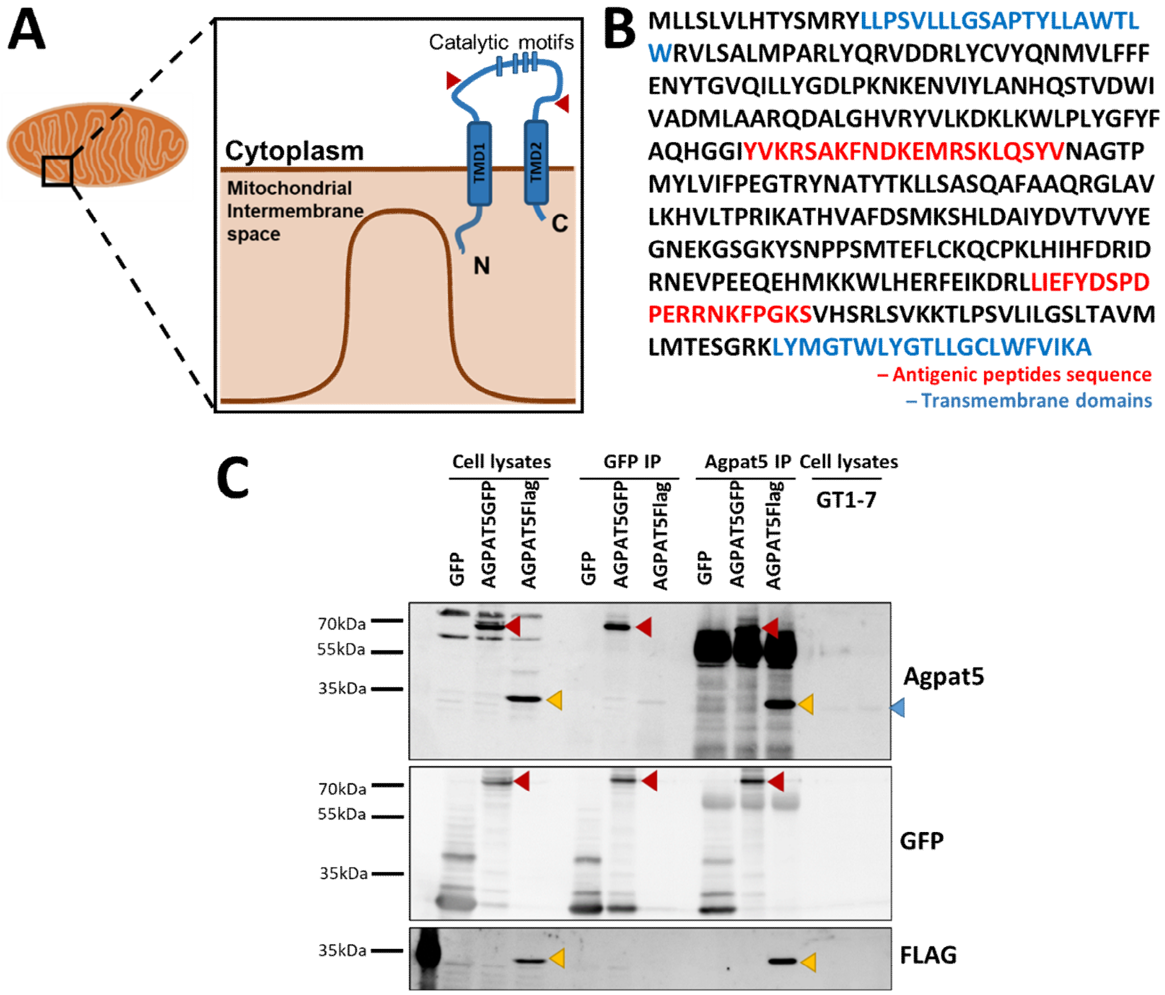

Supplementary Figure 3: Validation of affinity-purified anti-Agpat5 antibody

(A) Predicted membrane topology of murine Agpat5.

(B) Murine Agpat5 amino acid sequence. The two predicted transmembrane domains are in blue; the sequences of the peptides used for immunization of rabbits are in red.

(C) HEK293T cells were transfected with GFP, Agpat5GFP or Agpat5Flag constructs. Cell lysates were resolved on SDS-PAGE or first immunoprecipitated with mouse anti-GFP or rabbit anti-Agpat5 antibodies before separation by gel electrophoresis. The transfer membranes were then probed with rabbit anti-Agpat5, mouse anti-GFP or mouse anti-FLAG the antibodies. Representative immunoblotting image is presented. Red arrowheads: Agpat5-GFP; yellow arrowheads: Agpat5Flag; blue arrow: endogenous Agpat5.

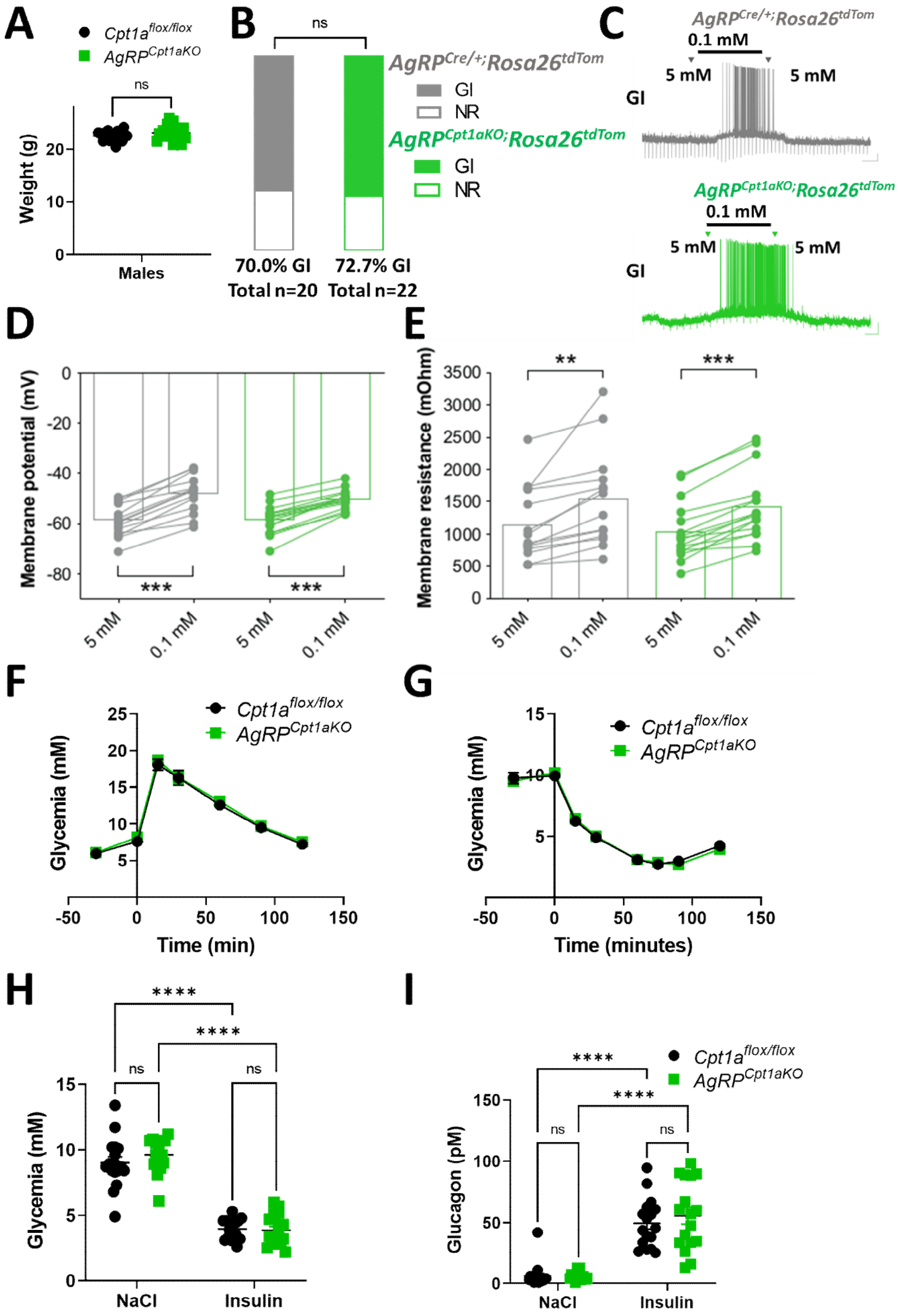

Supplementary Figure 4: *Cpt1a* inactivation in AgRP neurons does not alter glucose sensing or glucagon secretion in male mice

(A) Body weight of 8 weeks-old male *Cpt1a*$^{flox/flox}$ and *AgRP*^{CPT1aKO} littermate mice, n=17.

(B) Proportion of GI and NR AgRP neurons in *AgRP*$^{Cre/+;}$*Rosa26*^{tdTom} mice and *AgRP*$^{Cpt1aKO;}$*Rosa26*^{tdTom} mice. n=20-22 neurons/n=7-8 mice per genotype. Fisher's exact test.

(C) Patch clamp identification of AgRP GI neurons in *AgRP*$^{Cre/+;}$*Rosa26*^{tdTom} mice and *AgRP*$^{Cpt1aKO;}$*Rosa26*^{tdTom} mice.

(D) Changes in membrane potential in GI neurons activated by 0.1 mM glucose in *AgRP*$^{Cre/+;}$*Rosa26*^{tdTom} and *AgRP*$^{Cpt1aKO;}$*Rosa26*^{tdTom} male mice.

(E) Membrane resistance.

Data from n=18 neurons/n=8-10 mice per genotype. Before-after graphs display individual values. **p<0.01, *** p<0.001, two-tailed, paired Student's t-test.

(F) Glucose tolerance tests in *Cpt1a*$^{flox/flox}$ and *AgRP*^{Cpt1aKO} male mice.

(G) Insulin tolerance tests.

(H) Glycemia one hour after NaCl or insulin injection in *Cpt1a*$^{flox/flox}$ and *AgRP*^{Cpt1aKO} male mice.

(I) Plasma glucagon levels.

(F-I) Two independent cohorts, n=17 mice per genotype. Data are mean±SEM. ****p<0.0001, (F,G) two-way ANOVA (repeated measurements) with Sidak's multiple comparisons correction or (H,I) two-way ANOVA with Tukey's post hoc test.
