# Supplementary material for: Lipid biosynthesis enzyme Agpat5 in AgRP-neurons is required for insulin-induced hypoglycemia sensing and glucagon secretion
Source: Nat Commun. 2022 Sep 30;13:5761. doi: 10.1038/s41467-022-33484-6 (PMC9525695; doi:10.1038/s41467-022-33484-6)
Supplement: Supplementary file 3 — Reporting Summary [file 41467_2022_33484_MOESM3_ESM.pdf]

nature portfolio

Corresponding author(s): Bernard Thorens
bernard.thorens@unil.ch

Last updated by author(s): 13/5/22

Reporting Summary

Nature Portfolio wishes to improve the reproducibility of the work that we publish. This form provides structure for consistency and transparency in reporting. For further information on Nature Portfolio policies, see our Editorial Policies and the Editorial Policy Checklist.

Statistics

For all statistical analyses, confirm that the following items are present in the figure legend, table legend, main text, or Methods section.

n/a	Confirmed	
☐	☒	The exact sample size (*n*) for each experimental group/condition, given as a discrete number and unit of measurement
☐	☒	A statement on whether measurements were taken from distinct samples or whether the same sample was measured repeatedly
☐	☒	The statistical test(s) used AND whether they are one- or two-sided<br>*Only common tests should be described solely by name; describe more complex techniques in the Methods section.*
☒	☐	A description of all covariates tested
☐	☒	A description of any assumptions or corrections, such as tests of normality and adjustment for multiple comparisons
☐	☒	A full description of the statistical parameters including central tendency (e.g. means) or other basic estimates (e.g. regression coefficient) AND variation (e.g. standard deviation) or associated estimates of uncertainty (e.g. confidence intervals)
☐	☒	For null hypothesis testing, the test statistic (e.g. *F*, *t*, *r*) with confidence intervals, effect sizes, degrees of freedom and *P* value noted<br>*Give P values as exact values whenever suitable.*
☒	☐	For Bayesian analysis, information on the choice of priors and Markov chain Monte Carlo settings
☒	☐	For hierarchical and complex designs, identification of the appropriate level for tests and full reporting of outcomes
☒	☐	Estimates of effect sizes (e.g. Cohen's *d*, Pearson's *r*), indicating how they were calculated

Our web collection on statistics for biologists contains articles on many of the points above.

Software and code

Policy information about availability of computer code

Data collection	Doric software , 5.x(Tucker Davis Technologies) - fiber photometry<br>LabChart 8 software, 8v.1.21 (AD Instrument, Oxford, UK) - vagal nerve recordings<br>pClamp 10 data acquisition system , rel. 11.x (Molecular Devices) - electrophysiology<br>Wave software, v2.4.2 (Seahorse, Agilent) - Mitochondrial stress test<br>AxioVision, rel. 4.2 - immunohistochemstry image acquisition
Data analysis	PRISM GraphPad version 9.0<br>Jupyter interface (Python 3.0) , code available on GitLab (/pcanilho)<br>LabChart 8, v8.1.21- electrophysiology and vagal nerve recordings<br>ImageJ (Fiji), vrel. 2.4.0- immunoblotting analysis, immunohistochemistry analysis, electron microscopy analysis

For manuscripts utilizing custom algorithms or software that are central to the research but not yet described in published literature, software must be made available to editors and reviewers. We strongly encourage code deposition in a community repository (e.g. GitHub). See the Nature Portfolio guidelines for submitting code & software for further information.

Data

Policy information about availability of data

All manuscripts must include a data availability statement. This statement should provide the following information, where applicable:

- Accession codes, unique identifiers, or web links for publicly available datasets
- A description of any restrictions on data availability
- For clinical datasets or third party data, please ensure that the statement adheres to our policy

The accession number for the raw data (Fastq files) for hypothalamus RNAseq reported used in this study is Gene Expression Omnibus (GEO): GSE87586. The data sets for liver and white adipose tissue RNAseq in this study are GEO: GSE114845 and GEO: GSE79016, respectively.
Data supporting the findings of this work are available within the paper. Source data are available from the corresponding author on reasonable request.

Field-specific reporting

Please select the one below that is the best fit for your research. If you are not sure, read the appropriate sections before making your selection.

☒ Life sciences ☐ Behavioural & social sciences ☐ Ecological, evolutionary & environmental sciences

For a reference copy of the document with all sections, see nature.com/documents/nr-reporting-summary-flat.pdf

Life sciences study design

All studies must disclose on these points even when the disclosure is negative.

Sample size	For all experiments, the sample size was chosen based on power calculations using either two-sided T-test or a 2x2 factorial design (when appropriate). We also allowed for 10% surplus in the number of animals for the metabolic experiments to avoid losing statistical power in case if one or two animals met the exclusion criteria.<br>For metabolic cohorts we used 10-15 animals per group per independent cohort.<br>For hyperinsulinemic-hypoglycemic clamp we used 8 animals per group.<br>For AgRP cell number quantification we used three animals per group and for cFos immunostaining quantification we used 4-7 animals per group.<br>For fiber photometry we used 6 animals per group.
Data exclusions	Inadequate i.p. injection or health problem were used as exclusion criteria. One female and one male animal were excluded from the glucagon secretion during insulin-induced hypoglycemia experiment (due to an injection problem and a health problem respectively).
Replication	Key metabolic experiments (glucagon secretion during insulin-induced hypoglycemia were repeated in independent cohorts. All replication attempts were successful and showed the same phenotype.
Randomization	Littermate wild-type and knockout mice were randomly allocated to the treatment groups. To control for confining factors, body weight and number of animals per cage was kept the same between the treatment groups.
Blinding	Sample blinding was only performed during glucagon ELISA experiments (investigators were not blinded to group allocation during the ELISA procedure. For the electron microscopy data acquisition the experimenters were blinded by a different investigator. For the data collection for metabolic studies the investigators were blind to the genotype, but for practical reasons (injections and data collection performed by the same person that habituated the mice).

Reporting for specific materials, systems and methods

We require information from authors about some types of materials, experimental systems and methods used in many studies. Here, indicate whether each material, system or method listed is relevant to your study. If you are not sure if a list item applies to your research, read the appropriate section before selecting a response.

Materials & experimental systems

n/a	Involved in the study	
☐	☒	Antibodies
☐	☒	Eukaryotic cell lines
☒	☐	Palaeontology and archaeology
☐	☒	Animals and other organisms
☒	☐	Human research participants
☒	☐	Clinical data
☒	☐	Dual use research of concern

Methods

n/a	Involved in the study	
☒	☐	ChIP-seq
☒	☐	Flow cytometry
☒	☐	MRI-based neuroimaging

Antibodies

Antibodies used	Custom-generated, affinity-purified rabbit anti-Agpat5 antibody (BioTem, France);<br>Mouse anti-α-tubulin (T5168, Sigma-Aldrich, RRID:AB_477579);<br>Mouse anti-GFP (G6539, Sigma-Aldrich, RRID:AB_259941);<br>Mouse anti-FLAG (8146, Sigma-Aldrich, RRID:AB_10950495);<br>Donkey anti-rabbit (#926-32213, LI-COR, Cambridge, UK, RRID:AB_621848)<br>Donkey anti-mouse (#926-48072, LI-COR, Cambridge, UK), RRID:AB_2814906<br>Rabbit anti-c-fos (#2250, 1/1000, Cell Signaling, Danvers,USA), RRID:AB_2247211.
Validation	Anti-Agpat5 antibody was validated using in our laboratory (mouse Agpat5, mouse brain, murine hypothalamic cell lines and overexpressed murine Agpat5-Flag and Agpat5-GFP constructs in HEK23T cells. Commercially available antibodies were validated by the companies (for the mouse species and the applications used in our experiments) and tested using a positive control for each experiment in our laboratory prior to the use. In particular, c-fos staining was confirmed for every experiment using staining in PVT in the mouse brain as a positive control.

Eukaryotic cell lines

Policy information about cell lines

Cell line source(s)	GT1-7 cells (hypothalamic neuronal cell line, mouse, SV40 immortalised) (donation from Dr. Pamela L. Mellon's laboratory) (SCC116, Sigma Aldrich)<br>HEK293T cells were purchased from ATCC (Manassas, USA)
Authentication	GT1-7 cells (RRID:CVCL_0281)<br>HEK293T cells (RRID:CVCL_0063)<br>Cells were authenticated using morphological assessment and, for GT1-7 cell line, NeuropeptideY (NPY) expression was assessed, primers: forward_CCGCCACGATGCTAGGTAAC; reverse_CAGCCAGAATGCCCAAACAC).
Mycoplasma contamination	Routinely tested at the cell culture laboratory. All cells used in our experiments tested negative for mycoplasma.
Commonly misidentified lines<br>(See ICLAC register)	No commonly misidentified cell lines were used in this study.

Animals and other organisms

Policy information about studies involving animals; ARRIVE guidelines recommended for reporting animal research

Laboratory animals	Mice (purchased from genOway, Lyon, France or transferred under MTA from Prof. Peter Carmeliet laboratory (Katholieke Universiteit Leuven, Belgium) on C57BL6/N background). We used male mice aged between 5-7wo (for electrophysiology) and both male and female mice aged between 8-15wo for the metabolic studies. Mice housing conditions are described in the manuscript.
Wild animals	No wild animals were used in this study.
Field-collected samples	No field-collected samples were used in this study.
Ethics oversight	All procedures were conducted in accordance to the Swiss National Institutional Guidelines of Animal Experimentation (OExA; 455.163) with licenses approval (VD3363 and VD3686) issued by the Veterinary Office of Canton de Vaud (Vaud, Switzerland).

Note that full information on the approval of the study protocol must also be provided in the manuscript.
